# Removal of concentrated sulfamethazine by acclimatized aerobic sludge and possible metabolic products

**DOI:** 10.7717/peerj.1359

**Published:** 2015-11-03

**Authors:** Na Yang, Junfeng Wan, Shiju Zhao, Yan Wang

**Affiliations:** 1 School of Chemical Engineering and Energy, Zhengzhou University, Zhengzhou, China; 2 School of Civil and Environmental Engineering, Nanyang Technological University, Singapore, Singapore; 3 Advanced Environmental Biotechnology Centre, Nanyang Environment and Water Research Institute, Nanyang Technological University, Singapore, Singapore

**Keywords:** Acclimatized activated sludge, Adsorption, Biodegradation, Metabolites, Sulfamethazine (SMZ)

## Abstract

This article examined the biological removal of high concentrated sulfamethazine (SMZ) antibiotics by the acclimatized activated sludge in lab-scale SBRs system. The removal of SMZ was characterized by a quick adsorption and a slow process of biodegradation. The adsorption capacity of activated sludge for SMZ was 44 and 47 µg SMZ/g SS, respectively, with the initial SMZ concentrations of 1 and 2 mg/L. The adsorption process fitted pseudo-second-order kinetic model. In a series of batch studies, with the increase of initial SMZ concentration that were 1, 2, 3, 5, 7 and 9 mg/L, 56.0%, 51.3%, 42.2%, 29.5%, 25.0% and 20.8% of influent SMZ were biodegraded within 24 h of biological reaction, respectively. The Monod equation applied to simulate SMZ biodegradation had a good coefficient of determination (*R*2 > 0.99). Furthermore, the results of HPLC demonstrated that the SMZ was not completely removed by the acclimatized activated sludge. From the analysis of LC-MS, 4 intermediates of SMZ biodegradation were identified: Sulfanilic Acid, 4-amino-N-(4,6-dimethyl-2 pyrimidin) benzene sulfonamide, N-(4,6-dimethyl-2-pyrimidin)-4-N-(benzene sulfonamide) benzene sulfonamide, N-(4,6-dimethyl-2-pyrimidin)-4-N-(4,6-dimethyl pyrimidine) benzene sulfonamide, and N-(4,6-dimethyl-2-pyrimidin)-4-N-(3-dimethyl-4-N sodium benzene sulfonamide) benzene sulfonamide.

## Introduction

The environmental impact of residual pharmaceuticals and personal-care products (PPCPs) has become public concern due to their widespread occurrence in the environment (Gobel et al., 2005; McClellan & Halden, 2010; Vasiliadou et al., 2013). Among these emerging contaminants, antibiotics are extensively used not only in human and veterinary medicine but also as growth promoting agents in the modern farming and aquaculture industry (Garcia-Galan et al., 2011; Zhou et al., 2014). However, the presence of antibiotics and their metabolites in water can probably lead to the potential persistent danger to the ecological system and human health (Garcia-Galan, Silvia Diaz-Cruz & Barcelo, 2008).

Sulfonamides (SAs), as one group of typical antibiotics, are widely utilized in the livestock industry because of their low-cost and relative efficiency in the treatment of common bacterial infections (Perez-Moya et al., 2010; Perez-Trallero & Iglesias, 2003). The previous studies reported that the concentration of SAs in manure ranged from 10 µg/kg to 91 mg/kg (Christian et al., 2003; Jacobsen & Halling-Sorensen, 2006; Martinez-Carballo et al., 2007; Pfeifer et al., 2002). SAs residues have been detected in all kinds of environmental water matrix (Garcia-Galan, Silvia Diaz-Cruz & Barcelo, 2008). It is noted that the main entry route of SAs into soil environment was the land application of manure excreted by animals as well as biosolid from sewage treatment plants (Giger et al., 2003; Sukul & Spiteller, 2006). The environmental fate of SAs and their metabolites is currently unclear. The presence of both parent drugs and their respective metabolites has been considered together and demonstrated within the scope of an increasing number of research studies (Diaz-Cruz, Llorca & Barcelo, 2008; Gobel et al., 2004; Radke et al., 2009; Stoob et al., 2006). Sulfamethazine (SMZ), as one of the SAs, containing two ionizable functional groups: the aniline amine and the amide moieties (Gao & Pedersen, 2005). SMZ has been extensively used for many years in human therapy and the livestock industry (Braschi et al., 2010; Sarmah, Meyer & Boxall, 2006), is now frequently detected in both surface waters (Garcia-Galan et al., 2010) and groundwater in concentrations up to 2,482 ng/L and 220 ng/L (Batt, Snow & Aga, 2006), respectively. It is urgent and critical to develop an economical and reliable method for elimination of antibiotics in water and wastewater.

Generally, the physicochemical technologies are applied for pre-treatment of SMZ where the SMZ concentration are high. Physico-chemical processes yield a number of different intermediates or transformation products which, similarly to any metabolite, can be more stable than the parents and also have negative effects at the different trophic levels of the ecosystem (Trovo et al., 2009). Biological methods are inexpensive, no second pollution, simple to operate, and ecologically clean as compared with the physicochemical treatments. Activated sludge process represents one of the most intriguing microbial systems engineering for different specific purposes. Perez, Eichhorn & Aga (2005) investigated the fate of three SAs antibiotics sulfamethazine (SMZ), sulfamethoxazole and sulfathiazole at low concentration level (20 µg/L), found that about 50, 75, and 93% were removed via biodegradation in 10 days for these three antibiotics, respectively. Although several studies have reported that adsorption and biodegradation are important pathways accounting for antibiotic removal (Li & Zhang, 2010; Yang et al., 2011; Yang et al., 2012; Yin et al., 2014), little attention has been paid to their biological metabolic pathway and metabolites probably due to less concentration and analytical limitation (Huang et al., 2012; Garcia-Galan, Silvia Diaz-Cruz & Barcelo, 2008). Sequencing batch reactor (SBR) is most commonly employed in treating wastewater. But little is known about the mechanism for removal of SMZ in this process. Additionally, sequencing batch reactors (SBRs) process is relatively flexible and automatic to deal with different features of wastewater. Some lab-scale SBRs were performed to evaluate the biodegradability of PPCPs in wastewater (Perez, Eichhorn & Aga, 2005). Therefore, during the present study, concentrated SMZ was chosen as the targeted antibiotic which was fed to activated sludge over 6 months in order to enhance its biological removal capacity and investigate the possible metabolites.

Thus, the main objectives in this work were: (1) to evaluate the adsorption and biodegradation efficiency of concentrated SMZ by acclimatized activated sludge in a SBRs system; (2) to explore possible products of SMZ antibiotics through the identification of it metabolites by LC-MS.

## Materials and Methods

### Chemicals

The SMZ was purchased from Sigma-Aldrich Co (St. Louis, Missouri, USA), and the physico-chemical characteristics are shown in Table 1. All other chemicals were analytical grade and were purchased from Beijing Chemical Reagent Factory, China.

**Table 1:** Physico-chemical characteristics of SMZ.

Name	Chemical formula	Molecular weight (g/mol)	pKa	Melting point (°C)	Solubility (g/L)	Molecular structure
SMZ	C_12_ H_14_ N_4_ O_2_ S	278.33	2.65/7.65	197–200	1.5	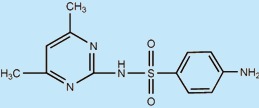

### Experimental set-up

Activated sludge was originally collected from the aeration tank in Wulongkou municipal wastewater treatment plant located in Zhengzhou, China. The sludge was acclimated in several lab-scale SBRs with synthetic wastewater. The lab-scale SBRs were an airlift column with a working volume of 2.0 L (internal diameter = 10 cm, height = 30 cm, H/D ratio = 3). The SBRs were operated with one cycle per day, and the cycle consisting of 1 h of influent feeding, 20 h of mixed aerobic phase, 2 h of sludge settling, 1 h of effluent withdrawal with a volumetric exchange ratio of 50%. Air was introduced through a fine-bubble diffuser at the bottom of the reactor. Gas flowmeter and electric valves were used to control the dissolved oxygen (DO) concentration more than 3 mg O_2_/L during aerobic period. The characteristics of synthetic wastewater were fed to SBRs system as following: the chemical oxygen demand (COD) 1,000 mg/L, SMZ 1–10 mg/L, Total nitrogen (TN) 50 mg N/L, TP 10 mg P/L. The SBR systems operated over 6 months. The concentration of SMZ was increased gradually each 30 days in the SBRs system in order to ensure the acclimatization effect of activated sludge. The operational conditions were 20 ± 1 °C and pH 7.5 ± 0.25.

### Batch study

After 6 months of lab-scale operation, the acclimatized activated sludge was then centrifuged and washed with deionized water several times until SMZ could not be detected. After the last centrifugation, the sludge was dissolved in distilled water and stored in a refrigerator at 4 °C. A series of batch tests was carried out with different initial concentration of SMZ (as the only external carbon source).

Two blank experiments, one with the reactor system containing SMZ without sludge and another containing sludge without SMZ, were carried out to ensure that no sorption of SMZ occurred on the reactors surface and no contamination of SMZ was introduced by the sampled sludge. The control experiments confirmed that self-hydrolysis, photodegration and adsorption on containers of SMZ could be neglected, which was also confirmed by Li & Zhang (2010). Thus, the self-hydrolysis, photodegration and adsorption on containers of SMZ could not be considered when using acclimatized activated sludge. Normally, sterilized activated sludge (through autoclave method) should be used as a control test for evaluation the adsorption and biodegradation effect, but this step would inevitably change its physico-chemical properties (structure, surface charge, etc.) of acclimatized activated sludge. Considering that the antibiotics adsorption by using activated sludge was a much fast process (Sukul & Spiteller, 2006), this study define that the decreased soluble SMZ in SBRs system for the first 2 h is mainly due to the adsorption through a series of the adsorption test, whereas the decreased soluble SMZ is due to the biodegradation effect after 2 h.

The sorption and degradation kinetic tests using acclimatized activated sludge were also done as contrast experiments. Before the adsorption experiment, the sludge was diluted to the required MLSS level with distilled water. Taking into account the concentration of SMZ in the environment is between ng/L and µg/L, the kinetic model for absorption was assessed only with respect to data obtained from absorption of lower concentrations. The sorption kinetic experiments were conducted in three 2 L SBRs with 1 L of mixed liquor. The SBRs were running simultaneously in continuous aerobic condition at 25 °C for 2 h with SMZ solution at 1 and 2 mg/L. Acclimatized activated sludge (MLSS = 10 g/L) was used at the beginning of the tests, and samples were collected from the SBRs at 0, 5, 15, 30, 60, 80, 100 and 120 min. In the biodegradation experiments, in order to obtain the specific SMZ biodegradation rates and identify metabolic intermediates of SMZ, the degradation tests were conducted with SMZ solution at various concentrations (1, 2, 3, 5, 7 and 9 mg/L) in the similar way used for the adsorption kinetics experiments. The samples were collected from the SBRs at 2, 3, 5, 7, 9, 12 and 24 h. The concentrations of residual SMZ were centrifuged at 10,000 rpm for 5 min, the supernatant was filtered through poly vinylidene difluoride filter membrane (0.22 µm) and stored at 4 °C until further analysis.

### Analytical methods

The COD concentration was determined by Hach Method 8000 with a DR5000 spectro photometer (Hach Co., Loveland, Colorado, USA). The MLSS concentration was analyzed in accordance with the Standard Methods (APHA, 2005). The analytical methods used for high performance liquid chromatography (HPLC) measurement of the aqueous samples were described previously (Xu et al., 2013). The contents of SMZ in water were determined by HPLC (P230II; Elite analytical instruments Co., Dalian, China) equipped with a 5 µm, 4.6 × 250 mm Agilent TC-C18 column and a UV detector at a wavelength of 267 nm. The mobile phase was a mixture of solution/acetonitrile 65:35 (v/v) at a flow rate of 1 mL/min. The column temperature was set at 30 °C. The detection limit of SMZ was determined to be 200 µg/L and this value was defined as the minimum concentration in the linear range with 3 times of the signal-to-noise ratio. Liquid chromatography–mass spectrometry (LC/MS, 6430 Triple Quad LC/MS; Agilent Technologies, Santa Clara, California, USA) was used to identify biological metabolic intermediates of SMZ. The analytical methods used for LC/MS measurement of the aqueous samples were described previously (Avisar, Lester & Mamane, 2010; Ben et al., 2014; Garcia-Galan et al., 2011; Mansour et al., 2014; Yang et al., 2011; Yang et al., 2012). An Agilent TC-C_18_ column (2.1 × 50 mm, 1.8 µm particle size) and a UV detector at a wavelength of 267 nm were employed. The mobile phase was a mixture of solution/methanol 80:20 (v/v) at a flow rate of 0.2 mL/min, the injection volume of the sample was 5 µL, the column temperature was set at 30°. The MS parameters were carried out with electrospray ionization in the positive ionization (ESI_+_) mode, and the operating conditions were as follows: desolvation gas flow 11 mL/min; source and desolvation temperatures 300 °C, respectively; capillary voltage 4,000 V; 135 V to obtain the fragmentation patterns when performing product ion scans. The applied collision gas was high purity nitrogen. For the MS analyses, MS data was obtained by scanning from m/z 50 to m/z 600.

## Results and Discussion

### Adsorption of SMZ by acclimatized activated sludge

**Figure 1:**
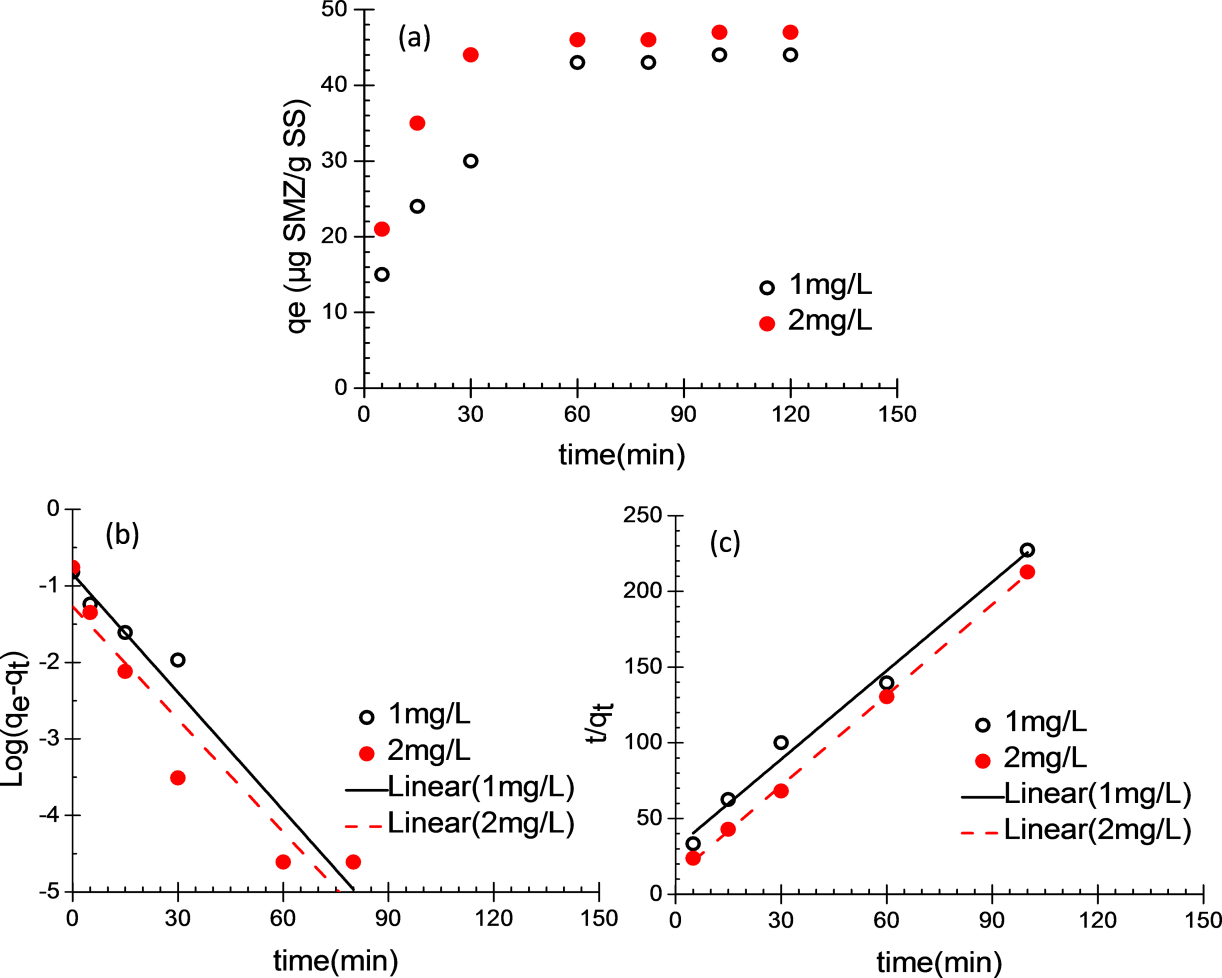
Batch adsorption of SMZ by acclimatized activated sludge. (A) The adsorbed SMZ by acclimatized activated sludge versus time; (B) pseudo-first-order adsorption kinetic plots and (C) pseudo-second-order adsorption kinetic plots; (MLSS = 10 g/L; 20 ± 1 ° C; pH 7.5 ± 0.25).

Figure 1A shows the variation of SMZ adsorbed by the unit mass of acclimatized activated sludge according to time at different initial concentration (1 and 2 mg/L). The pseudo-first-order and pseudo-second-order kinetic models were applied to simulate the SMZ adsorption (Sag & Aktay, 2002). The pseudo-first-order model is expressed as follows:

(1) \begin{eqnarray*} \log \left({q}_{e}-{q}_{t}\right)={\log q}_{e}-\left(\frac{{k}_{1}}{2.303}\right)t \end{eqnarray*}

where *k*_1_ is the pseudo-first-order rate constant (min^−1^) of adsorption, *q_e_* and *q_t_* (µg SMZ/g SS) are the amounts of SMZ adsorbed at equilibrium and time *t*, respectively.

The adsorption mechanism can probably be described as pseudo-second-order model, and it is expressed as follows:

(2) \begin{eqnarray*} \frac{t}{{q}_{t}}=\frac{1}{{k}_{2}{q}_{e}^{2}}+\left(\frac{1}{{q}_{e}}\right)t \end{eqnarray*}

where *k*_2_ is the pseudo-second-order rate constant of adsorption (g/(mg ⋅ min)), *q_e_* and *q_t_* (µg SMZ/g SS) are the amount of SMZ adsorbed at equilibrium and time *t*, respectively.

In this study, SMZ uptake was calculated using the following equation:

(3) \begin{eqnarray*} q t=\frac{{C}_{0}-{C}_{t}}{\mathrm{MLSS}} \end{eqnarray*}

where *q_t_* (µg SMZ/g SS) is the amount of adsorbed SMZ at time *t*; *C*_0_ (µg/L) and *C_t_* (µg/L) are the initial and residual SMZ concentrations at time *t*, respectively. During 2 h of batch tests, the unit adsorption capacity of SMZ on acclimatized activated sludge increased with the adsorption time extended. The adsorption reached saturation at 60 min, the adsorption equilibrium was established and the unit adsorption capacity of SMZ (*q_e_*) was respectively 44 and 47 µg SMZ/g SS, when initial SMZ concentration were 1 and 2 mg/L (Fig. 1A). In this study, pseudo-first-order kinetic model and pseudo-second-order kinetic model were used to fit the experimental data. Figure 1B and Table 2 were presented that the rate constant *k*_1_ was calculated and the values were 0.0842 and 0.1183 (min^−1^), the *q*_*e*,*cal*_ was calculated and the values were 39.32 and 42.93 µg/g, the correlation coefficients (*R*^2^) were 0.946 and 0.899, respectively. As shown in Fig. 1C and Table 2, the rate constant *k*_2_ was calculated and the values were 0.169 and 0.3832 g/(mg ⋅ min), the *q*_*e*,*cal*_ was calculated and the values were 45.33 and 47.76 µg/g, the correlation coefficients (*R*^2^) were 0.989 and 0.999. In the pseudo-second-order expression, the correlation coefficients of determination (*R*^2^ > 0.98) and *q*_*e*,*cal*_ (45.33 and 47.76 µg/g) was closed to *q*_*e*,*exp*_ (44.02 and 47.01 µg/g), these results indicate that the assumption of pseudo-second-order kinetic model is appropriate for describing the adsorption of SMZ onto acclimatized activated sludge. These results suggest that the sorption rate was probably controlled by chemical sorption involving valence forces or covalent forces between SMZ and the activated sludge.

**Table 2:** Pseudo-first-order and pseudo-second-order kinetic parameters for the SMZ adsorption by acclimatized activated sludge.

		Pseudo-first-order			Pseudo-second-order		
SMZ (mg/L)	*q*_*e*,exp_ (**μ** g/g)	*q*_*e*,cal_ (**μ** g/g)	*k*_1_ (min^−1^)	*R* ^2^	*q*_*e*,cal_ (μ g/g	*k*_2_ g/(mg⋅ min)	*R* ^2^
1	44.02	39.32	0.0842	0.946	45.33	0.169	0.989
2	47.01	42.93	0.1183	0.899	47.76	0.3832	0.999

### Degradation of SMZ by acclimatized activated sludge

**Figure 2:**
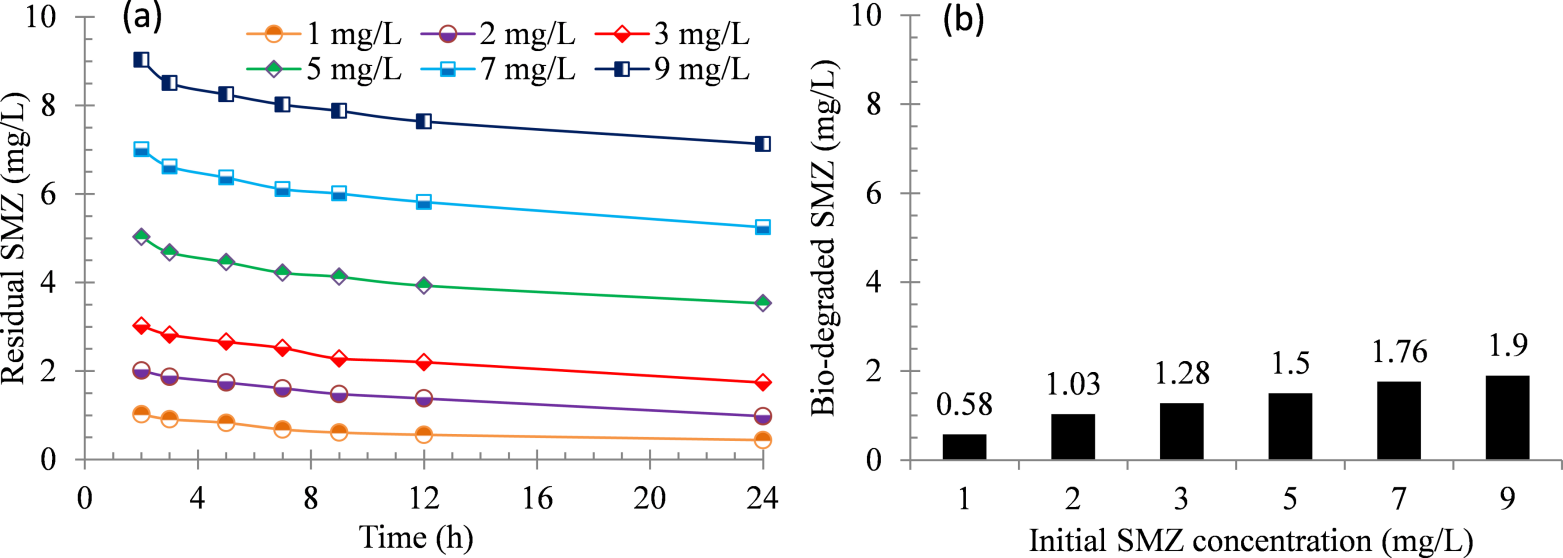
Biodegradation of SMZ by acclimatized activated sludge at different initial concentrations. (A) Residual SMZ during 24 h of reaction and (B) bio-degraded SMZ after 24 h of reaction.

The results show that the SMZ adsorption by activated sludge is believed as the first and rapid step (Fig. 1A) and the soluble SMZ is biologically removed via biodegradation during 24 h of reaction after 1 h of the adsorption step. The profiles of SMZ concentrations over time during 24 h of batch test are presented in Fig. 2. The concentration of SMZ in supernatant is measured as function of time. The SMZ removal rates are 56.0%, 51.3%, 42.2%, 29.5%, 25.0% and 20.8% when the initial SMZ concentrations are 1, 2, 3, 5, 7 and 9 mg/L, respectively. The result shows an existence of a lag phase before the onset of antibiotics biodegradation, this is corresponding to the previous studies (Plosz, Leknes & Thomas, 2010; Yang et al., 2011), it can be explained that the biodegradation starts when sulfonamides have fully established sorption equilibrium with the activated sludge; or the micro-organisms prefer to utilize readily biodegradable substrates before the degradation of the antibiotics. And this may be why a lag phase appeared in this study.

**Figure 3:**
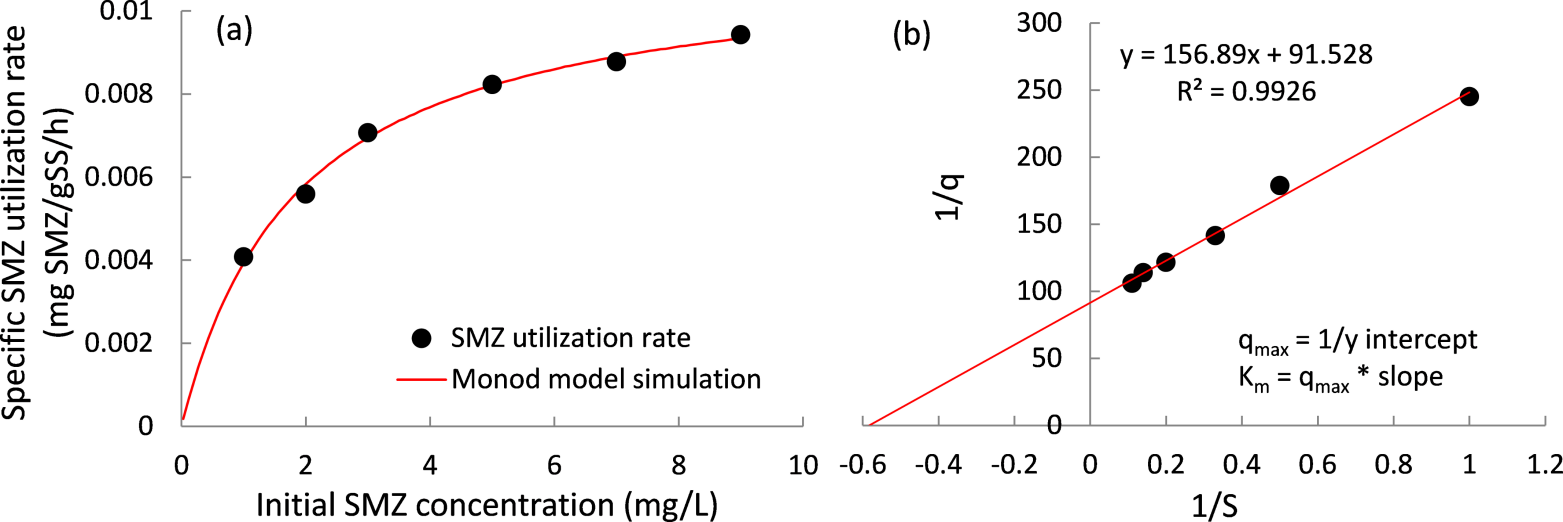
SMZ biodegradation kinetics. (A) Specific SMZ utilization rates as a function of initial SMZ concentration and (B) utilization rate data fit to the Lineweaver–Burk plot to estimate the Kinetic parameters Km and qobs, max.

The specific SMZ biodegradation rates at various SMZ concentrations were determined in this study (Fig. 3), and a Monod-type equation (Eq. (4)) was employed to fit the experimental data.

(4) \begin{eqnarray*} {q}_{\mathrm{obs}}=\frac{{q}_{\mathrm{obs},\max }[S]}{{K}_{m}+[S]} \end{eqnarray*}

where *q*_obs_ and *q*_obs,max_ are the specific SMZ utilization rate and its maximum value (mg SMZ/(g SS ⋅ h)). [*S*] is the concentration of SMZ (mg SMZ/L) and *K_s_* is the half-saturation coefficient (mg SMZ/L).

The Monod model is introduced to describe the degradation rates of SMZ at different initial concentrations. The experimental data can be well described by Eq. (4), evidenced by the *R*^2^ values higher than 0.99, and the maximum specific SMZ utilization rate (*q*_obs,max_) is found to be 0.0113 ± 0.0003 mg SMZ/g SS/ h.

### Microbial degradation products of SMZ

**Figure 4:**
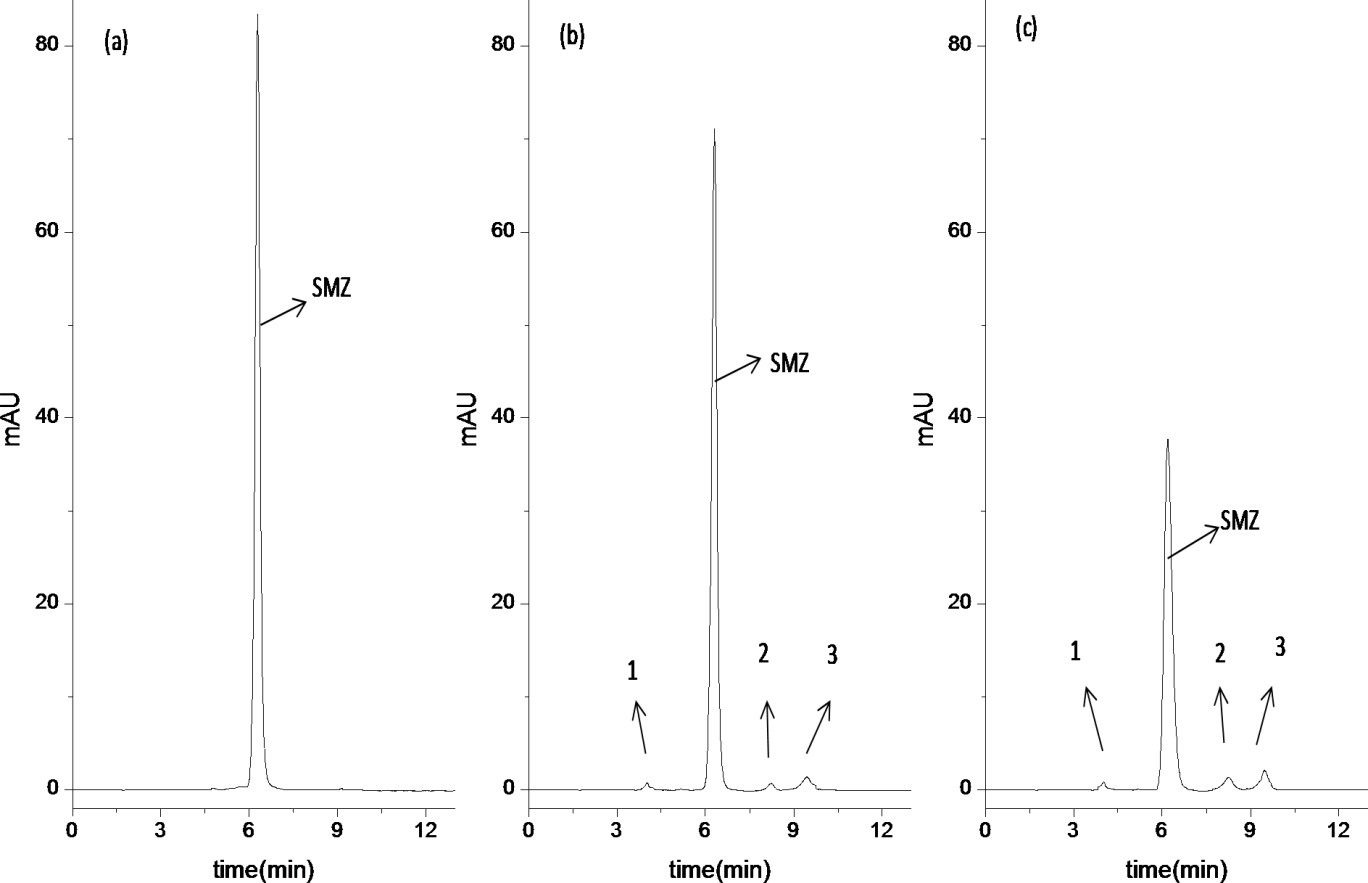
Analysis of SMZ degradation in bioreactor as function of time by HPLC. (A) 5 min of reaction, (B) 8 h of reaction, (C) 24 h of reaction.

As shown in Fig. 4A, the sample extracted from the reactor at 5 min was detected through HPLC. One big chromatographic peak can be observed and confirmed as SMZ. Compared with the HPLC diagram of sample extracted from SBRs system at 5 min, the HPLC analysis of samples extracted from the reactor at 8 h and 24 h of reaction shows that the peak of SMZ (Figs. 4B and 4C) still remains but reduces, whereas three new chromatographic peaks (1, 2 and 3) appear. Table 3 confirmed that relative concentration of four chromatographic peaks. peak 1, SMZ, 2 and 3 were 0.095, 9.5, 0.12 and 0.29 mg/L at 8 h of reaction, respectively, peak 1, SMZ, 2 and 3 are 0.16, 9.1, 0.33 and 0.45 mg/L at 24 h of reaction, respectively, when initial SMZ’s concentration was 10 mg/L. A few factors, such as adsorption, mineralization and detection limitation, may cause quantities and types of intermediate products less detected.

As shown in Figs. 4B and 4C, three peaks are probably the metabolic intermediates of SMZ, which indicates that SMZ is partially biodegraded by acclimatized activated sludge (Garcia-Galan et al., 2011). Although the HPLC results can be used as an effective method to quantify the variation of metabolic intermediates, the metabolic intermediates of SMZ are difficult to distinguish. In order to further analyze metabolic intermediates of SMZ, LC-MS analysis is used to identify the possible main products of SMZ.

**Table 3:** The relative concentration of SMZ and intermediate products.

Peak	Peak height		Characteristic peak area		Relative concentration	
	8 h	24 h	8 h	24 h	8 h	24 h
1	0.71	0.89	9.40	13.74	0.0095	0.016
SMZ	71.09	37.75	939.93	776.83	0.95	0.91
2	0.70	1.36	11.63	27.98	0.012	0.033
3	1.39	2.12	28.64	38.59	0.029	0.045

### Investigation of the metabolic products of SMZ by LC-MS

**Figure 5:**
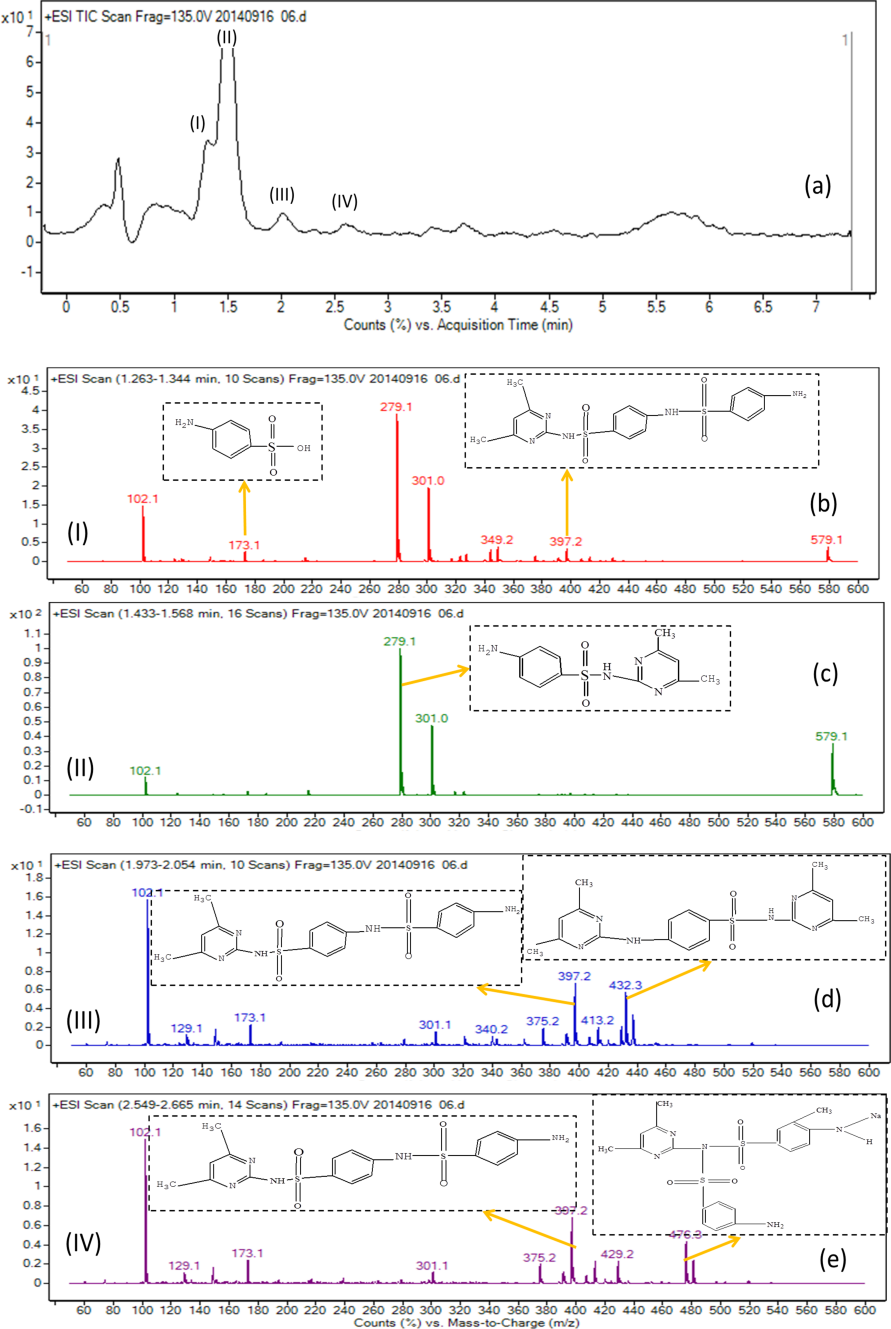
Mass spectra of the degradation products of SMZ after biodegradation.

In order to elucidate the mechanism of biological degradation of SMZ, the identification of the main reaction intermediates was carried out by LC-MS analysis (Fig. 5A) and by comparing their retention time and their molar mass (Table 4). As it is shown in Fig. 5A and Table 4, 4 absorption peaks are respectively determined at 1.2–1.3 min, 1.4–1.6 min, 1.9–2.1 min and 2.5–2.7 min after 24 h processing, in which the second (1.4–1.6 min) absorption peak was known as SMZ absorption peak which corresponded to m/z of 279.1, 301.0 and 579.1 (Fig. 5C and Table 4). Its elemental compositions were elucidated as SMZ + H^+^, SMZ + Na^+^ and 2SMZ + Na^+^. This demonstrated that sludge didn’t completely degrade SMZ.

A first peak appeared at a chromatographic retention time of 1.2–1.3min, which corresponded to m/z of 173.1 and 397.2 (Fig. 5B and Table 4). Its elemental compositions were elucidated as SA and N4,6D2P4NBSBS. The third peak appeared at a chromatographic retention time of 1.9-2.1 min, which corresponded to m/z of 173.1, 397.2 and 432.3 (Fig. 5D and Table 4). Its elemental compositions were elucidated as SA, N4,6D2P4NBSBS and N4,6D2P4N4,6DPBS. The fourth peak appeared at a chromatographic retention time of 2.5–2.7 min, which corresponded to m/z of 173.1, 397.2 and 476.3 (Fig. 5E and Table 4). Its elemental compositions were elucidated as SA, N4,6D2P4NBSBS and N4,6D2P4N3D4NSBSBS. SA agreed with previous studies in which activated sludge treatment by combination with electro Fenton for the mineralization of SMZ, SA was defined as intermediate metabolite (Mansour et al., 2012). N4,6D2P4NBSBS, N4,6D2P4N4,6DPBS and N4,6D2P4N3D4NSBSBS were still present the parent product with other groups. According to the retention time and molar mass of SMZ and its metabolite, we could propose that SA, N4,6D2P4NBSBS, N4,6D2P4N4,6DPBS and N4,6D2P4N3D4NSBSBS were the main degradation products.

**Table 4:** Proposed main sulfamethazine metabolites.

Chemicals	Molecular structure	Molecular mass (g mol^−1^)	Reference
Sulfanilic acid (SA)	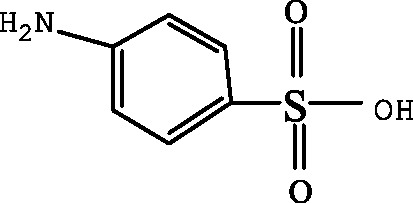	173.1	Mansour et al. (2012)
4-amino-N-(4,6-dimethyl-2-pyrimidin) benzene sulfonamide (SMZ + H^+^)	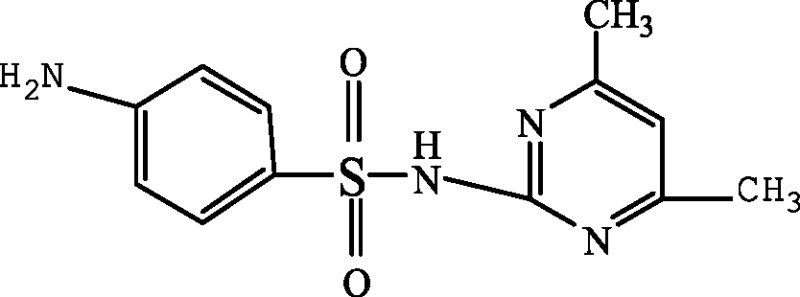	279.1	Garcia-Galan et al. (2011)
N-(4,6-dimethyl-2-pyrimidin)-4-N-(benzenesulfonamide) benzenesulfonamide (N4,6D2P4NBSBS)	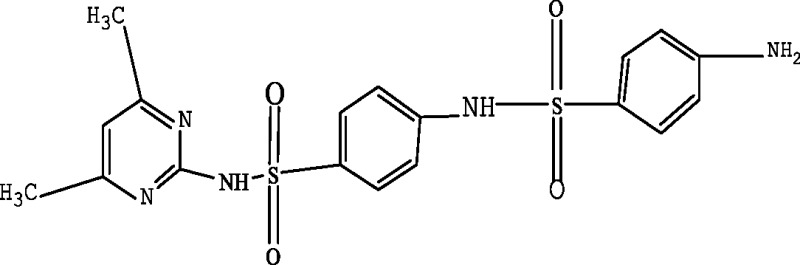	397.2	Garcia-Galan et al. (2011)
N-(4,6-dimethyl-2-pyrimidin)-4-N-(4,6-dimethyl pyrimidine) benzene sulfonamide (N4,6D2P4N4,6DPBS)	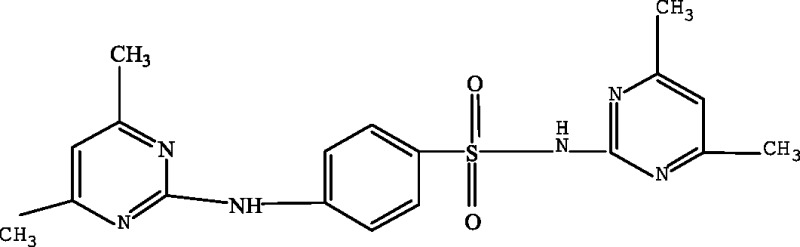	432.3	Garcia-Galan et al. (2011)
N-(4,6-dimethyl-2-pyrimidin)-4-N-(3-dimethyl-4-N-sodium -benzenesulfonamide) benzene sulfonamide (N4,6D2P4N3D4NSBSBS)	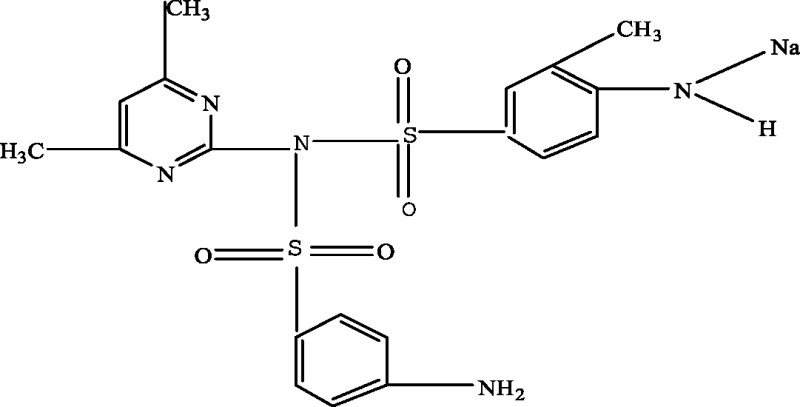	476.3	Garcia-Galan et al. (2011); Garcia-Galan, Silvia Diaz-Cruz & Barcelo (2008)

## Conclusions

The equilibrium reached after 1 h of the adsorption for acclimatized activated sludge with different initial concentration of SMZ. The adsorption capacity was 44 and 47 mg SMZ/g SS, respectively, when initial SMZ concentration was 1 and 2 mg/L. The SMZ biodegradation rate accelerated when initial concentration of SMZ increased from 1 to 9 mg/L. According to its metabolite retention time and molar mass, several metabolites were elucidated. The results proved that acclimatized activated sludge could be efficiently used as an alternative option to remove concentrated SMZ in industrial wastewater.

## Supplemental Information

10.7717/peerj.1359/supp-1 Supplemental Information 1 Raw data
